# Updated ACMG/AMP specifications for variant interpretation and gene curations from the ClinGen RASopathy expert panels

**DOI:** 10.1016/j.gimo.2025.103430

**Published:** 2025-04-17

**Authors:** Emma H. Wilcox, Ryan F. Webb, Kezang C. Tshering, Madeline Y. Hughes, Hélène Cavé, Marina T. DiStefano, Hannah Dziadzio, Kate Garber, Bruce D. Gelb, Karen W. Gripp, Shoji Ichikawa, Jennifer A. Lee, Hannah McCurry, Marco Tartaglia, Bradley Williams, Martin Zenker, Lisa M. Vincent, Heather Mason-Suares, ClinGen RASopathy Expert Panel, Bradley Williams, Bruce Gelb, Hannah Dziadzio, Heather Mason-Suares, Hélène Cavé, Jennifer Lee, Karen Gripp, Kat Lafferty, Kezang Tshering, Lisa Vincent, Luis Enrique Gomez, Marco Tartaglia, Marina DiStefano, Martin Zenker, Reza Ahmadian, Ryan Webb, Shoji Ichikawa

**Affiliations:** 1 Medical and Population Genetics Program, Broad Institute of MIT and Harvard, Cambridge, MA; 2 The Warren Alpert Medical School of Brown University, Providence, RI; 3 Département de Génétique, Hôpital Robert Debré (AP-HP), and Université Paris-Cité, Paris, France; 4 Department of Human Genetics, Emory School of Medicine, Atlanta, GA; 5 Mindich Child Health and Development Institute, Icahn School of Medicine at Mount Sinai, New York, NY; 6 Division of Medical Genetics, Nemours Children’s Hospital, Wilmington, DE; 7 Ambry Genetics, Aliso Viejo, CA; 8 Greenwood Genetic Center, Greenwood, SC; 9 Ospedale Pediatrico Bambino Gesù, IRCCS, Rome, Italy; 10 GeneDx, Gaithersburg, MD; 11 Institute of Human Genetics, University Hospital Magdeburg, Magdeburg, Germany; 12 Natera Inc, Austin, TX; 13 Mass General Brigham, Laboratory for Molecular Medicine, Cambridge, MA

**Keywords:** ClinGen, Noonan, Ras/MAPK, RASopathy, Variant interpretation

## Abstract

**Purpose:**

The ClinGen RASopathy (RAS) Variant Curation Expert Panel (VCEP) previously established RASopathy specifications to the American College of Medical Genetics and Genomics (ACMG) and Association of Molecular Pathology (AMP) variant classification framework for more consistent and accurate variant classification. Advances in the understanding of RASopathies and new clinical genetic testing algorithms required updated specifications.

**Methods:**

The RAS Gene Curation Expert Panel recurated 6 gene-disease relationships, and the RAS VCEP evaluated the previous specifications to develop updated RASopathy specifications for the ACMG/AMP framework. The performance of these updated specifications was tested by reassessing 59 previously classified variants and 88 new pilot variants.

**Results:**

Five gene-disease relationships were upgraded to Definitive, whereas 1 was upgraded to Moderate. Updated specifications were applied to 11 ACMG/AMP criteria for disorders with a dominant inheritance, 3 criteria for recessive inheritance, and 4 criteria to align with recommendations from the ClinGen Sequence Variant Interpretation Working Group. Assessment of variants demonstrated no major shifts in classifications compared with previous RAS VCEP or ClinVar classifications.

**Conclusion:**

Updated RASopathy specifications improve the classification of variants associated with recessive disease and observed in exome/genome cases. Most of these specifications may also be used as a baseline for other rare Mendelian disorders.

## Introduction

RASopathies constitute a group of conditions caused by dysregulation of the highly conserved Ras/mitogen-activated protein kinase pathway. The prevalence of RASopathies has been estimated as 1:1000, with Noonan syndrome (NS; OMIM 163950) being the most common.^1^ Other RASopathies include NS with multiple lentigines (formerly LEOPARD syndrome; OMIM 151100), Costello syndrome (OMIM 218040), cardio-facio-cutaneous syndrome (OMIM 115150), NS with loose anagen hair (also known as Mazzanti syndrome; OMIM 607721), NF1 (OMIM 613113), Legius syndrome (OMIM 611431), and CBL (Cbl proto-oncogene) syndrome (OMIM 613563). Characteristic features include distinctive craniofacial gestalt/dysmorphism, webbed neck, cardiovascular abnormalities (most commonly pulmonary valve stenosis and hypertrophic cardiomyopathy [HCM]), short stature, variable cognitive deficits, musculo-skeletal defects, and ectodermal abnormalities.^1^ Because of variation in severity and overlapping clinical presentation, distinguishing between these conditions can be difficult.^2^ Therefore, genetic testing is important to solidify the clinical diagnosis.

Accurate molecular diagnostics require standardization of the strength of gene-disease relationships and clinical variant interpretations across clinical laboratories. However, interpretations vary widely between laboratories.^3^^,^^4^ Multiple efforts are underway to address this issue.^5^ For example, the Clinical Genome Resource (ClinGen; www.clinicalgenome.org) established a framework for classifying gene-disease relationship strength^6^ and the American College of Medical Genetics and Genomics (ACMG) with the Association of Molecular Pathology (AMP) published guidelines for standardized variant interpretation.^7^ ClinGen established Gene Curation Expert Panels (GCEPs) to curate gene-disease relationships and Variant Curation Experts Panels (VCEPs) to create disease-specific specifications based on ACMG/AMP guidelines.^8^ The RASopathy (RAS) GCEP previously curated 44 gene-disease relationships^9^ and the RAS VCEP outlined specifications for evaluating variants in 9 RASopathy-associated genes.^10^

Since the adoption of ACMG/AMP recommendations, it has become apparent that additional specifications and guidance are required.^3^^,^^11^^,^^12^ Therefore, the ClinGen Sequence Variant Interpretation (SVI) Working Group provided additional recommendations for clinical variant interpretation.^13^ To align RASopathy specifications with these new SVI recommendations and ensure that past specifications can be applied to recently associated RASopathy genes, the RAS VCEP refined variant interpretation rule specifications including the following: (1) restructuring how cases are counted toward PS2/PM6, PS4, BS2, and BP2/BP5 using a point system; (2) clarifying use of prenatal cases for PS4 and PS2/PM6; (3) defining phenotypic criteria needed to apply BS2; (4) clarifying use of PM1 and PM5; (5) adjusting computational predictor cutoffs for PP3 and BP4; and (6) evaluating AR specific rules in the context of RASopathies.

## Materials and Methods

### RASopathy GCEP and VCEP

The RAS VCEP and GCEP are international groups of medical geneticists, laboratory diagnosticians/scientists, and research scientists with various areas of expertise.^10^ All RAS VCEP and GCEP members are required to disclose potential conflicts of interest to ClinGen.

Genes evaluated by the RAS GCEP are available at https://search.clinicalgenome.org/kb/affiliate/10021?page=1&size=25&search=

Variants curated by the RAS VCEP are available at https://erepo.clinicalgenome.org/evrepo/ui/summary/classifications?columns=ep&values=RASopathy%20VCEP&matchTypes=exact&pgSize=25/

### Evaluation of gene-disease relationships

Prior gene-disease relationships, classified as Limited through Strong in Grant et al,^9^ were re-evaluated following the ClinGen Recuration Procedure.^6^^,^^14^ Recurations were completed for (1) *LZTR1* (HGNC:6742) and autosomal dominant (AD) NS, (2) *LZTR1* and autosomal recessive (AR) NS, (3) *MRAS* (HGNC:7227) and AD NS, (4) *PPP1CB* (HGNC:9282) and AD NS with loose anagen hair, (5) *SOS2* (HGNC:11188) and AD NS, and (6) *RRAS2* (HGNC:17271) and AD NS. Genes with a Definitive gene-disease relationship to at least 1 RASopathy in Grant et al^9^ were not recurated. Primary recuration was completed by a ClinGen biocurator using only published data following the ClinGen Gene Clinical Validity Curation Process Standard Operating Procedure (SOP) version 7 for *LZTR1/*AD NS and *PPP1CB*, version 8 for *SOS2,* and version 9 for *MRAS*, *RRAS2*, and *LZTR1/*AR NS (https://clinicalgenome.org/curation-activities/gene-disease-validity/documents/). Under versions 7 and 8, case-level evidence was awarded the recommended default points. Under version 9, de novo missense variants were awarded the recommended default 0.5 points. If a missense variant had functional data supporting a gain-of-function (GoF) mechanism, it was awarded 1 point. De novo missense variants with functional data supporting a GoF mechanism were awarded the maximum 1.5 points. Functional evidence was awarded the recommended default points for each SOP version. The evidence for each gene-disease relationship was approved by the RAS GCEP as described in Gelb et al^10^

### Updates to ACMG/AMP criteria

Each ACMG/AMP criterion was reviewed for new or updated RASopathy specifications based on recommendations by the ClinGen SVI Working Group (https://clinicalgenome.org/working-groups/sequence-variant-interpretation/), new disease mechanisms (ie, AR NS), newly curated genes, or issues arising during RAS VCEP curation of variants in previously curated RASopathy-associated genes. Updated specifications are listed in Table 1 and were approved by the ClinGen SVI Working Group. Specifications not listed in Table 1 can be referenced in Gelb et al.^10^ To determine the final variant classification, the Richards et al^7^ combination rubric of criteria was used.

**Table 1:** Revised pathogenic and benign American College of Medical Genetics and Genomics and Association of Molecular Pathology modified criteria for the RASopathies

Pathogenic		
Criteria	Criteria Description	Specification
PVS1	Only applicable to *LZTR1*	Disease specific
PS1	May use analogous residues and splice site variants	Disease specific
PS2/PM6	Increase strength for multiple observations; see Table 3 for point-based scoring	Disease specific
PS4	Increase strength for multiple observations; see Table 5 for point-based scoring	Disease specific
PM2_P	For *LZTR1*, 0.0025%; PM2 no longer applicable	Strength
PM1	Applicable to defined functional domains. May be applied in conjunction with PM5.	Disease specific
PM5	PM5_S applicable when ≥5 case counts across ≥2 different substitutions have been reported at a codon.	Strength
PM3	Only applicable to *LZTR1*	Disease specific
PP2	Only applicable to genes with a missense *z*-score ≥ 3.09 in gnomAD: *BRAF*, *MAP2K1*, *PTPN11*, and *PPP1CB*	Disease specific
PP3	REVEL ≥ 0.7	Disease specific

Benign		
Criteria	Criteria Description	Specification
BS2	Increase strength for multiple observations; see Table 7 for point-based scoring	Strength
BP2	Increase strength for multiple observations; see Table 9 for point-based scoring	Strength
BP4	REVEL ≤ 0.3	Disease specific
BP5	Increase strength for multiple observations; see Table 9 for point-based scoring	Strength

*REVEL*, Rare exome variant ensemble learner.

### Determining rare exome variant ensemble learner score thresholds for PP3 and BP4 application

The rare exome variant ensemble learner (REVEL) is a computational tool incorporating predictions from other algorithms for missense variants.^15^ To improve systematic variant review, REVEL score thresholds of ≥0.7 and ≤0.3 recommended by the ClinGen SVI for application of PP3 (ie, deleterious effect predicted by computational evidence) and BP4 (ie, no impact predicted by computational evidence), respectively, were assessed for applicability to RASopathy-associated variants.^16^ These thresholds were evaluated using a subset of variants previously classified as pathogenic (P; *n* = 40) or benign (B; *n* = 32) by the VCEP in 9 genes: *PTPN11* (HGNC:9644; *n* = 14), *BRAF* (HGNC:1097; *n* = 12), *SOS1* (HGNC:11187; *n* = 17), *KRAS* (HGNC:6407; *n* = 5), *RAF1* (HGNC:9829; *n* = 9), *HRAS* (HGNC:5173; *n* = 5), *MAP2K1* (HGNC:6840; *n* = 3), *MAP2K2* (HGNC:6842; *n* = 4), and *SHOC2* (HGNC:15454; *n* = 3). For inclusion in this analysis, P variants required at least 1 Very Strong criterion or 2 Strong criteria in addition to other Moderate and/or Supporting criteria. B variants required either BA1 (ie, occurring at a much higher population frequency than expected) or 2 Strong criteria. For each variant category, the average REVEL score, SD, 95% CI, and lower and upper bound cutoffs were calculated and compared with SVI recommended thresholds. Subsequently, analysis was performed on 43 additional P variants, 24 likely pathogenic (LP) variants, 24 likely benign (LB) variants, and 5 B variants in the 9 genes classified by the RAS VCEP before November 2019. A *t* test was performed to compare the average REVEL scores of the original 40 P variants with additional P/LP variants and the original 32 B variants with additional B/LB variants.

### Determining functional domains and hotspots for PM1 and PM5_Strong application

Functional domains for PM1 application were determined by mapping domains reported in the literature in conjunction with cluster analysis of contiguous regions with a minimum of 5 P residues and lacking B missense variation. Moreover, gene subfamilies (eg, RAS genes) were aligned using the National Center for Biotechnology Information's (NCBI) constraint-based multiple alignment tool (https://www.ncbi.nlm.nih.gov/tools/cobalt/re_cobalt.cgi) to identify shared functional domains and hotspots.

Hotspot locations for PM5_Strong application were determined by comparing the number of probands with the observed P/LP variants at the same amino acid position in the literature and large reference laboratory ClinVar submissions relative to common B/LB (BA1/BS1 met) variation reported in gnomAD v2.1.1. A predetermined residue hotspot for PM5_Strong application was defined as a residue with at least 2 different P/LP changes, without B variation, observed across a total of at least 5 probands. PM5 application remains as defined in Gelb et al.^10^ Note, the type of residue change was considered not relevant because its impact is captured during each variant’s individual classification process through the application of PP3/BP4 from in silico prediction tools (ie, REVEL).

### In-frame insertions/deletions and repetitive area assessments using PM4 and BP3

To provide clarity on PM4 usage (ie, protein length changes due to in-frame deletions/insertions in a nonrepeat region or stop-loss variants) and BP3 (ie, in-frame deletions/insertions in a repetitive region without a known function), each gene was scanned for repetitive elements within its coding regions by viewing all available tracks within the Repeats track of the University of California Santa Cruz (UCSC) Human Genome Browser (http://genome.ucsc.edu) (GRCh37/hg19; accessed 1 April 2023). Impact assessment was based on each gene-disease mechanism. Pathogenic repetitive variation has not been previously reported in the RASopathy genes.^17^

### Performance of updated specifications and variant classification

To test the performance of these updates, 59 variants previously classified in Gelb et al^10^ were reassessed. These variants represented the *BRAF* (*n* = 7), *RAF1* (*n* = 8), *MAP2K1* (*n* = 5), *MAP2K2* (*n* = 7), *PTPN11* (*n* = 6), *SHOC2* (*n* = 6), *SOS1* (*n* = 6), *KRAS* (*n* = 8), and *HRAS* (*n* = 6) genes. An additional 88 pilot variants in *LZTR1* (*n* = 23), *MRAS* (*n* = 7), *NRAS* (HGNC:7989; *n* = 15), *PPP1CB* (*n* = 8), *RRAS2* (*n* = 5), *RIT1* (HGNC:10023; *n* = 15), and *SOS2* (*n* = 15) were assessed to further test the performance of these specifications on new genes. Published data and internal laboratory data were used for variant curation. All variant classifications have been uploaded into ClinVar.

## Results

### Gene-disease relationships

Of the recurated gene-disease relationships (*n* = 6), 5 were upgraded to Definitive and 1 to Moderate (Table 2) based on case-level evidence and time elapsed since the first proposal of the relationship. Despite the biological interaction of MRAS with the Ras/mitogen-activated protein kinase pathway and the SHOC2-MRAS-PP1C protein holophosphatase complex,^18^ lack of clinical data resulted in Moderate gene-disease validity. Of note, for all Moderate relationship assessments like *MRAS,* the highest clinical classification a variant can receive is LP.^19^

**Table 2:** Updated gene-disease relationship strength

Gene	Noonan (Points)	NS-LAH (Points)
*LZTR1* (AD)	Definitive (13)	-
*LZTR1* (AR)	Definitive (12.5)	-
*PPP1CB*	-	Definitive (12.5)
*SOS2*	Definitive (12.5)	-
*MRAS*	Moderate (9)	-
*RRAS2*	Definitive (12.5)	-

Scored points for each relationship are shown in parentheses.

*AD*, autosomal dominant; *AR*, autosomal recessive; *NS-LAH*, Noonan syndrome with loose anagen hair.

### Updated ACMG/AMP specifications

Additional recommendations are needed for previously unaccounted for scenarios (eg, prenatal cases with limited phenotypes), changes in common testing strategies (ie, variants detected during exome/genome sequencing), and for new RASopathy disease inheritance patterns. In addition, such specifications must incorporate new recommendations from the ClinGen SVI Working Group. Therefore, all new SVI recommendations were reviewed to determine if they were applicable to the RASopathies, and the following were updated: (1) reducing PM2 (ie, absent in population databases) to a supporting level to avoid placing excessive weight on absence or rarity of variants (https://clinicalgenome.org/site/assets/files/5182/pm2_-_svi_recommendation_-_approved_sept2020.pdf), (2) mirrored point-based scoring of PS2/PM6 (ie, de novo occurrences) SVI recommendations for specific criteria (see below for details), (3) changes in applying PS3 (ie, functional data; see below for details), and (4) limiting the application of PP2 (ie, missense variant in a gene that has a low rate of benign missense variation) to genes with a gnomAD missense *z*-score greater than 3.09, indicating significant missense constraint.^13^ Only *BRAF*, *MAP2K1*, *PTPN11*, and *PPP1CB* have missense *z*-scores greater than 3.09; therefore, PP2 can only be applied to these genes (see Supplemental Table 1 for *z*-scores).

#### Point-based scoring: PS2/PM6, PS4, BS2, and BP2/BP5

The ClinGen SVI Working Group recommends using a point-based system to determine the strength of de novo evidence (PS2 and PM6) based upon 3 parameters: parental relationships status (genetic testing), phenotypic consistency, and number of de novo observations (https://www.clinicalgenome.org/site/assets/files/3461/svi_proposal_for_de_novo_criteria_v1_1.pdf). The RAS VCEP adapted this recommendation and applied this framework to case observation related codes PS4, BS2, BP5, and BP2. For PS2 (ie, de novo with confirmed parental relationships) and PM6 (ie, assumed de novo), the RAS VCEP incorporated 3 categories of phenotypes: consistent with a RASopathy, limited, or not consistent. The number of points awarded to each case is based on phenotypic consistency and whether parentage is confirmed or assumed (Table 3). A clinical diagnosis for determination of phenotype consistency with a RASopathy must be performed by a medical geneticist or specialist experienced in RASopathies. Prenatal cases are restricted to a limited phenotype category and must display at least 1 of the following nonspecific, but overlapping, RASopathy phenotypes: HCM, increased nuchal translucency, cystic hygroma, pleural effusions, or hydrops.^20^, ^21^, ^22^ Other scenarios included in the limited phenotype category are clinical RASopathy next-generation sequencing gene panel cases with no clinical information provided and exome/genome cases with a phenotype consistent with a RASopathy, such as pulmonary valve stenosis and/or HCM in conjunction with short stature. Probands with nonconsistent phenotypes are typically identified on exome/genome sequencing and should not be awarded any points for PS2/PM6.

**Table 3:** De novo occurrences in a proband(s) with the disease and no family history

Phenotypic Consistency	Points Per Proband	
	Confirmed De Novo (PS2)	Assumed De Novo (PM6)
Phenotype is consistent with a RASopathy^a^	2	1
Limited phenotypic information^b^	1	0.5
Phenotype not consistent with RASopathy	0	0

a Exclusive of prenatal cases.

b Applicable to prenatal cases, cases with a clinical order of a RASopathy panel without clinical information, and cases with limited clinical information in other global tests (such as exome sequencing). Phenotypes for prenatal cases include hypertrophic cardiomyopathy, increased nuchal translucency, cystic hygroma, or hydrops.

Once all PS2 and PM6 probands are scored, the combined overall score determines the strength of PS2/PM6 applied (Table 4). All evidence for a variant should combine into usage of only 1 final de novo code of PS2 or PM6, with preference to PS2 if parentage is confirmed for any proband. For example, if there is a de novo variant in a proband with a clinical diagnosis of a RASopathy and parentage is confirmed (2 points) and 2 de novo probands with a clinical diagnosis of a RASopathy by a medical geneticist and parentage is not confirmed (2 points), the overall score is 4 points and PS2_VeryStrong is applied for that variant.

**Table 4:** ACMG/AMP evidence strength level for de novo occurrence(s)

Supporting (PS2_Supporting or PM6_Supporting)	Moderate (PS2_Moderate or PM6)	Strong (PS2 or PM6_Strong)	Very strong (PS2_VeryStrong or PM6_VeryStrong)
0.5 points	1 point	2 points	4 points

*ACMG*, American College of Medical Genetics and Genomics; *AMP*, Association of Molecular Pathology.

A similar but slightly modified point-based scoring system was applied to PS4 (ie, variant observed in affected individuals for a dominant disorder). For this criterion, 4 phenotypic categories were determined: (1) consistent with a RASopathy, (2) limited, (3) no clinical information or only nonspecific features, and (4) well phenotyped but consistent with a non-RASopathy disorder. The consistent and limited categories are the same as defined above and awarded 1 or 0.5 points per proband, respectively (Table 5). Cases with no clinical information or isolated features associated with high genetic heterogeneity (eg, intellectual disability, attention deficit hyperactivity disorder, and isolated cardiomyopathy) are awarded no points, whereas well-phenotyped cases that are consistent with non-RASopathy disorder (eg, clinical diagnosis of Cornelia de Lange syndrome [CdLS]) are awarded −1 point per proband. BP5 (ie, variant found in a case with an alternate molecular basis for disease) may also be applicable for these cases (see below). This last scenario typically occurs during exome/genome testing. Once all probands are scored, the total overall score determines the strength applied (Table 6).

**Table 5:** Phenotypic information to apply PS4

Phenotypic Consistency	Points Per Proband
Individual well phenotyped with features of a RASopathy	1
Limited phenotypic information compatible with RASopathy^a^	0.5
No clinical information or isolated clinical features	0
Well phenotyped but consistent with non-RASopathy disorder^b^	−1

a Applicable to prenatal cases, cases with a clinical order of a RASopathy panel without clinical information, and cases with limited clinical information in other global tests (such as exome sequencing). Phenotypes for prenatal cases include hypertrophic cardiomyopathy, increased nuchal translucency, cystic hygroma, or hydrops.

b Negative points for PS4 represent proband affected with a non-RASopathy congenital disorder rather than a healthy individual (BS2). This typically applies to probands tested by exome analysis with multiple other clinical features supporting a distinct syndromic disorder (e.g., CHARGE, CdLS).

**Table 6:** ACMG/AMP evidence strength level for applying PS4

Supporting (PS4_Supporting)	Moderate (PS4_Moderate)	Strong (PS4)
1.0 point	3.0 points	5.0 points

*ACMG*, American College of Medical Genetics and Genomics; *AMP*, Association of Molecular Pathology.

The point-based scoring for BS2 (ie, observed in a healthy adult individual) includes 4 phenotypic categories (Table 7) and typically applies to relatives tested during segregation studies. A healthy individual is awarded −3 points if homozygous and −1 point if heterozygous. When the only phenotypic information provided is unaffected, only −0.25 points should be awarded. When no clinical information is provided or only nonspecific phenotypes (see above) are noted, no points should be awarded. Once all healthy individuals are scored, the total overall score determines the strength applied (Table 8).

**Table 7:** Phenotypic information to apply BS2

Phenotypic Consistency	Points Per Individual
Healthy homozygous individual assessed for a RASopathy	−3
Healthy heterozygous individual assessed for a RASopathy	−1
No phenotypic information other than unaffected heterozygote^a^	−0.25
No clinical information or nonspecific clinical features	0

a Typically applicable to parental or sibling samples during clinical family evaluations.

**Table 8:** ACMG/AMP evidence strength level for applying BS2

Supporting (BS2_Supporting)	Moderate (N/A)	Strong (BS2)
−1 point	N/A	−3.0 points

*ACMG*, American College of Medical Genetics and Genomics; *AMP*, Association of Molecular Pathology; *N/A*, not applicable.

In addition to applying a modified point system, the RAS VCEP clarified the usage of BP5 (ie, variant found in a case with an alternate molecular basis for disease) to include cases with a clinical suspicion or diagnosis of non-RASopathy disorder and an identified causative LP/P variant. This scenario typically occurs during exome/genome testing. For example, −1 point can be applied to a variant in a RASopathy-associated gene identified in a case with CdLS and a disease-causing *NIPBL* (HGNC:28862) variant (Table 9). If no causative *NIPBL* (or other CdLS-associated gene) variant was identified, no points should be applied.

**Table 9:** Information to apply BP5/BS2

Phenotypic Consistency	Points Per Individual
Phenotype inconsistent with a RASopathy and causative variant has been identified, -or- Molecular cause of a RASopathy is identified in a different RASopathy gene, -or- Molecular cause of a RASopathy is identified in trans or cis with the variant being classified	−1
Phenotype inconsistent with a RASopathy and no causative variant identified/reported	0

Points should be additive between BP5 and BP2 (ie, variant found in cis/trans with LP/P variant), and only 1 code should be applied (Table 10). Of note, these codes should be applied cautiously as rare compound (ie, in trans in the same gene) and double (ie, variants in different genes) heterozygous cases have been reported.^23^^,^^24^

**Table 10:** ACMG/AMP evidence strength level for applying BP5/BP2

Supporting (BP5/BP2)	Moderate (N/A)	Strong (BP5_Strong/ BP2_Strong)
−1 point	N/A	−3.0 points

*ACMG*, American College of Medical Genetics and Genomics; *AMP*, Association of Molecular Pathology; *N/A*, not applicable.

#### REVEL scores: PP3 and BP4

**Figure 1:**
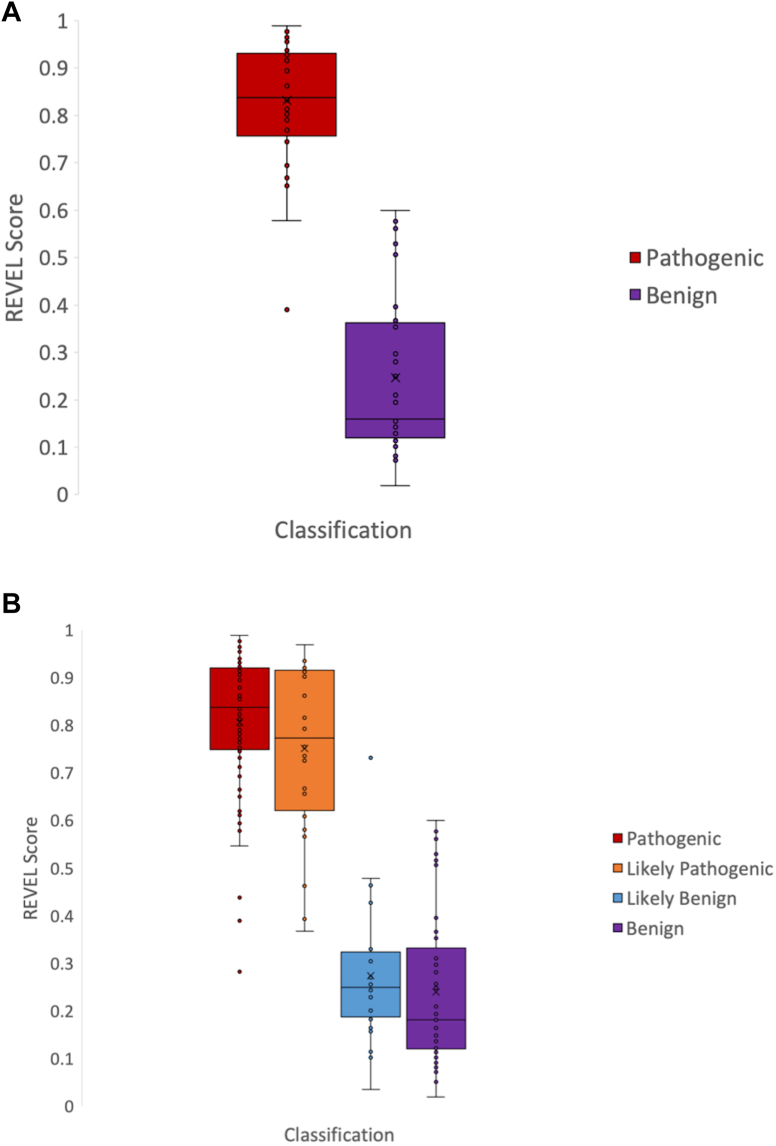
**Rare exome variant ensemble learner (REVEL) scores of variation in 9 RASopathy genes.** A. Box plot of the REVEL scores for 40 pathogenic and 32 benign variants. See Materials and Methods for number of variants in each gene. B. Box plot of the REVEL scores for 83 pathogenic, 24 likely pathogenic variants, 24 likely benign variants, and 37 benign variants.

The ClinGen SVI recommends using 1 of 4 suggested computational prediction tools, including REVEL, for codes PP3/BP4.^16^ REVEL predicts the pathogenicity of missense variants based on a combination of scores from 13 individual prediction tools, allowing for a more systematic way to aggregate multiple tools.^15^ To determine if the ClinGen SVI recommended thresholds (ie, ≤0.3 for BP4 and ≥0.7 for PP3) for the supporting criteria^16^ were applicable to RASopathy-associated variants, the REVEL scores for 40 P and 32 B variants were assessed (Figure 1A). The average REVEL score for P variants was 0.83 (SD = 0.12; 95% CI 0.79-0.87). Conversely, the average REVEL score for B variants was 0.25 (SD = 0.17, 95% CI 0.19-0.31). This analysis was then extended to include an additional 67 P/LP and 29 B/LB variants (Figure 1B). Student *t* tests showed that there was no significant difference between the average REVEL score for the 40 initial P variants and the additional 67 P/LP variants (*P* = .69) or the 32 initial B variants and the additional 29 B/LB variants (*P* = .53). Including these additional variants, 99% of P/LP variants were excluded at a REVEL score of ≤0.3, and 98.5% of B/LB variants were excluded at a REVEL score of ≥0.7. Therefore, it was determined that the ClinGen SVI recommended thresholds for supporting criteria^16^ were applicable to RASopathy-associated variants. The RAS VCEP does not recommend increasing the strength applied to PP3/BP4 at this time because of the nature of the algorithms favoring a loss-of-function (LoF) over a GoF disease mechanism.^15^

#### Assessing functional assays: PS3 and BS3

The ClinGen SVI Working Group has published additional requirements for applying PS3 (ie, functional evidence supporting a damaging effect).^25^ Review of the existing functional data in the literature and comparing them with these requirements led to capping PS3 at a moderate strength. PS3_Supporting may be applied if a variant has the expected abnormal results in one of the approved assays (see Supplemental Table 2 for more details). PS3_Moderate may be applied if a variant has the expected abnormal results in at least 2 different assays. Because well-known P variants have been observed to have wild-type results in these assays dependent on basal conditions, stimuli, or cell lines used,^26^, ^27^, ^28^, ^29^ BS3 is not applicable. Of note, there are currently no approved functional assays for *PPP1CB.*

#### In-frame insertions/deletions and repetitive areas: PM4 and BP3

No benign repetitive areas were identified in any RASopathy-associated gene; therefore, BP3 (ie, in-frame deletions/insertions in a repetitive region without a known function) is not applicable. On the contrary, PM4 (ie, protein length changes because of in-frame deletions/insertions in a nonrepeat region) remained applicable based on its original primary definition in Richards et al.^7^ An example of an appropriate PM4 application is *RRAS2* NC_000011.10:g.14358797_14358805dup NM_012250.6:c.70_78dup p.(Gly24_Gly26dup). This variant represents a duplication of the coding region glycine 24 through glycine 26 and is consistent with protein length change due to in-frame deletions/insertions in a nonrepeat region.

#### Paralogous genes: PS1

Many of the RASopathy-associated genes are in gene families: RAF (*BRAF* and *RAF1*), RAS (*HRAS*, *KRAS*, *MRAS*, *NRAS*, *RIT1*, and *RRAS2*), MAP2K (*MAP2K1* and *MAP2K2*), and SOS (*SOS1* and *SOS2*). Because the genes within these families often share analogous residues, the RAS VCEP previously specified PS1 to apply when the same amino acid change, either within the same gene or at an analogous position in a gene family member, has been previously classified as P.^10^ For example, PS1 can be applied to the *MRAS* NC_000003.12:g.138397333C>T NM_001085049.3:c.203C>T p.(Thr68Ile) variant because this change corresponds to the *HRAS* NC_000011.10:g.533883G>A NM_005343.4:c.173C>T p.(Thr58Ile) variant. Each gene family was aligned (Supplemental Figures 1-4) to aid in application of PS1.

PS1 may also be applied to splice site variants at the same location as a known P variant in the same or analogous gene position. The ClinGen SVI Splicing Subgroup recommendations, including splice region definitions and PS1 strength, should be applied.^30^ It is important to confirm that the known P splicing variant and the variant being classified have the same predicted splice impact using the same in silico tools, if RNA evidence is not available. Because RASopathies are typically associated with GoF variation, prediction of the impact of the splicing event on the protein is crucial. For example, an in-frame splicing event is likely to result in the production of a truncated protein that may result in a GoF mechanism. Alternatively, an out-of-frame splice variant is expected to result in nonsense-mediated decay and/or protein truncation, which is most consistent with LoF. Of note, splice variants within the canonical splice positions should also be considered for application of PVS1 in genes associated with LoF (see AR specifications section).

#### Refining hotspots and functional domains: PM1 and PM5

Because there is significant overlap between codes PM5 (ie, another P variant at the same location) and PM1 (ie, hotspot and functional domains), it was previously decided that these codes could not be combined.^10^ However, in practice, it can be difficult to determine which code to apply, and they are often used incorrectly.^11^^,^^12^ We recommend that PM5/PM5_Strong is used for hotspots and PM1 is used for predefined, well-established functional domains. PM5 and PM1 may now be used in conjunction; however, PM5_Strong cannot be applied with PM1.

Well-defined functional domains (PM1) were assessed for the 7 new RASopathy-associated genes: *MRAS*, *NRAS*, *RIT1*, *RRAS2*, *SOS2*, *LZTR1*, and *PPP1CB.* The RAS gene family has 4 functional domains that qualify for PM1: the P-Loop, switch 1 region (SW1), switch 2 region (SW2), and SAK motif.^31^, ^32^, ^33^, ^34^ All RAS family members contain the first 3 domains; however, *MRAS*, *RIT1*, and *RRAS2* lack the SAK domain (Supplemental Figure 2). No well-defined functional domains were observed for *SOS1*/*SOS2*, *LZTR1*, or *PPP1CB*; therefore, PM1 cannot be used for these genes.

Hotspots were predetermined for the usage of PM5_Strong by using current data from ClinVar, publications, and gnomAD v2.1.1. PM5_Strong was assigned when at least a total of 5 probands exhibited at least 2 or more different P/LP changes at that position. Over 15 PM5_Strong hotspots were identified using this approach (Supplemental Table 1). No PM5_Strong hotspots were identified in the *LZTR1*, *PPP1CB*, *MAP2K1*, *MAP2K2*, *RAF1*, or *SHOC2* genes. This is not an exhaustive list for PM5_Strong, and its application should be evaluated over time. PM5/PM5_Strong may be applied to analogous residues. PM5 is not applicable to variants that alter splicing.

#### AR specifications

New specifications are required to account for AR NS, which is typically associated with biallelic LoF/hypomorphic missense variants in *LZTR1*.^35^ The RAS VCEP assessed all ACMG/AMP criteria for AR NS and recommends usage of PVS1 (ie, LoF) and PM3 (ie, variant in trans observed in affected individuals for a recessive disorder) following ClinGen SVI guidelines^36^ (https://clinicalgenome.org/site/assets/files/3717/svi_proposal_for_pm3_criterion_-_version_1.pdf). For PM3 usage, points should be reduced by half for any proband with limited phenotypic information (see PS4). See Supplemental Figure 5 for PVS1 decision tree.

The current population frequency thresholds for BA1 and BS1 for AD and AR RASopathy are GroupMax (Grpmax) filtering allele frequencies of ≥0.0005 and ≥0.00025, respectively. Using gnomAD v2.1.1, these thresholds were revalidated by reviewing the frequency of all known P/LP AD RASopathy variants. Then applicability of these thresholds to AR NS was determined by assessing the frequency of all known P/LP AR *LZTR1* variants. To account for the heterozygote status of AR disease, application of PM2_Supporting for AR *LZTR1* variants requires a higher threshold of <0.000025. Of note, population frequency thresholds for noncontinental populations (ie, bottlenecked populations) may need to be manually assessed by applying a 95% confidence interval in the cardiodb allele frequency app (“calculate AF” tab at https://www.cardiodb.org/allelefrequencyapp/) as previously described.^37^

### Classifying novel variants in genes with multiple disease mechanisms

**Figure 2:**
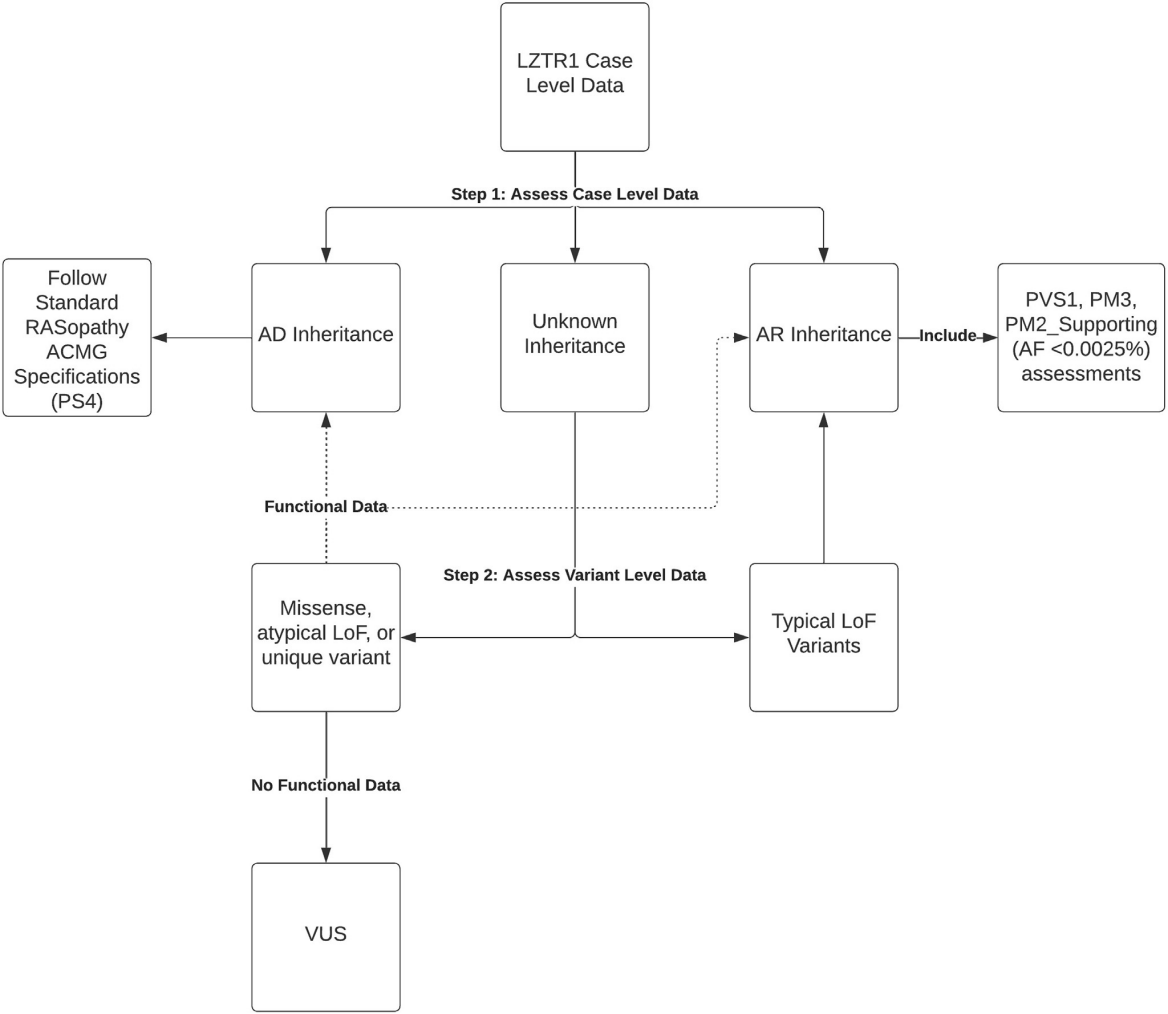
**Decision tree for applying autosomal recessive or autosomal dominant guidelines to *LZTR* variants.** ACMG, American College of Medical Genetics and Genomics; AD, autosomal dominant; AF, allele frequency; AR, autosomal recessive; LoF, loss-of-function; VUS, variant of uncertain significance.

Classifying variants in *LZTR1* is more complex than in other RASopathy-associated genes because variants may cause dominant or recessive disease. AD NS is typically associated with dominant-negative missense *LZTR1* variants, whereas AR NS is associated with LoF and/or hypomorphic *LZTR1* variants.^35^^,^^38^ To determine which criteria to apply to a novel variant, start by reviewing case-level data to establish an inheritance pattern. If a clear inheritance pattern can be determined, use the corresponding criteria. If a pattern is not immediately clear, presumed LoF variants should be assessed using specifications for AR NS (ie, PVS1, PM3, and PM2_Supporting). For all other variants, functional data (eg, p-ERK, p-MEK, LZTR1 stability, and LZTR1 subcellular localization), if available, may guide which criteria to apply. If functional data are unavailable, and there is no clear inheritance pattern, the variant remains a variant of uncertain significance (VUS) (Figure 2).

### Performance of updated specifications in variant classification

Overall, 147 variants were assessed using these updated specifications. Variants were split among 2 groups: 59 variants previously classified by the RAS VCEP using the original specifications and 88 pilot variants in the newer RASopathy genes. Of the 59 previously classified variants, 32 had a previous classification of P/LP, and 27 had a previous classification of VUS or B/LB. Using the original classification data, 44% (14/32) of the P/LP variants would have downgraded classification to LP or VUS when applying these updated specifications. However, new clinical case data retained the original classification in all except for 3 variants, which did downgrade from P to LP because of a reduction of PS3 (ie, functional data) to PS3_Moderate or PS3_Supporting. Of the other 11 variants that would have downgraded without new or updated case evidence, reduction of PM2 (ie, absent in population databases) strength to PM2_Supporting would account for 6 variants, reduction of PS3 to PS3_Moderate or PS3_Supporting for 4 (total 7, including those that did change above), and a reduction of both PM2 and PS3 for 1. New clinical case data also resulted in 3 LP variants upgrading to P. Limiting PS3 to a Moderate level and PM2 to a supporting level did affect variant classification on the pathogenic spectrum.

Of the 27 previously classified VUS or B/LB variants, only 1 LB variant would have changed classification to VUS because of the removal of BS3 (ie, functional data). However, newly available computational data retained its classification as LB. New data also resulted in 1 VUS downgrading to LB and 1 LB variant downgrading to B. Variant classification on the benign spectrum is not significantly changed by these updated specifications, which is expected because many of these variants were classified using our original general population frequency thresholds (ie, BA1/BS1).^10^

The performance of these updated specifications was then evaluated by classifying 88 pilot variants in the newer RASopathy genes (see Materials and Methods). At the time of evaluation, 48 variants had at least 1 classification of P/LP, 5 were classified as VUS, and 35 had at least 1 classification of B/LB in ClinVar. Of the 48 ClinVar variants with at least 1 classification of P/LP, 27 variants were classified as P, 17 as LP, and 4 as VUS using the updated specifications. All 5 ClinVar VUS maintained a VUS classification using the updated specifications. Of the 35 ClinVar variants with at least 1 classification of B/LB, 21 variants were classified as B, 7 as LB, and 7 as VUS using the updated specifications. All B variants and 57% of LB variants used codes BA1/BS1 (ie, high allele frequency in the general population). All variants, except 1, which changed classification from LB/B/LP/P/ to VUS had discrepant classifications in ClinVar, including at least 1 VUS classification. The 1 exception was only classified by OMIM. These results demonstrate that there was no egregious shift in classifications (eg, B to/from P) when applying the updated specifications.

## Discussion

Updated specifications were created to provide additional guidance for variants identified during exome/genome sequencing and for new disease mechanisms and inheritance patterns. Specific RASopathy-associated genes were intentionally excluded, notably *NF1* (HGNC:7765) and *SPRED1* (HGNC:20249). Specifications for these genes are currently under development by the ClinGen Neurofibromatoses and Schwannomatosis VCEP (https://www.clinicalgenome.org/affiliation/50114/). Until those specifications are available, our specifications may be applied to those genes and used as a framework for other rare Mendelian disorders with a frequency of less than 1:1000.

Most RASopathy-associated genes are also associated with hematologic malignancy, most notably juvenile myelomonocytic leukemia and some solid tumors. Cancer-associated somatic variants typically have a stronger GoF activity than those observed in germline disease.^39^ Many of these somatic variants may be embryonic lethal,^39^ and rare cases of early lethality/severe neonatal presentations support this.^40^^,^^41^ Because the underlying disease mechanism for the germline and somatic variants is the same, using data from somatic variants for criteria in classifying germline variants was considered. However, most P somatic hotspots have corresponding P germline hotspots.^42^ For example, recurrent somatic variant *PTPN11* NC_000012.12:g.112450406G>A NM_002834.5:c.226G>A p.(Glu76Lys) is at the same location as recurrent germline variant *PTPN11* NC_000012.12:g.112450408G>T NM_002834.5:c.228G>T p.(Glu76Asp).^42^ Therefore, it was determined that specifying criteria for somatic data in these guidelines was not required, and general recommendations for applying somatic data for germline classification would suffice.^43^

Although there were no major shifts in classifications when applying the updated specifications, limiting the strength of PS3 (ie, functional data) affected the ability to classify rare variation on the pathogenic spectrum. In our original study, we determined that 54% of well-established P variants relied on PS3 for P classification.^10^ When determining how the new ClinGen SVI recommendations^25^ could affect RASopathy-associated variant interpretation, the RAS VCEP performed a comprehensive analysis of all published functional data and identified gaps, specifically in the number of available controls for these studies. This resulted in capping PS3 at a Moderate level. To remove this limitation, the RAS VCEP has initiated a collaboration with researchers to define and perform the additional functional studies necessary so that this criterion may once again reach a Strong level.

These updated specifications give greater clarity when classifying RASopathy-associated variants, especially those identified during exome and genome sequencing. The RAS VCEP will continue to refine these specifications as newer disease mechanisms and testing strategies evolve, curate discrepant variants in ClinVar, and collaborate with researchers to ameliorate PS3 usage.

## Data Availability

All variant data were submitted to ClinVar under Organization ID 506439: https://www.ncbi.nlm.nih.gov/clinvar/submitters/506439/.

## Members of the ClinGen RASopathy Expert Panel

Bradley Williams, Bruce Gelb, Hannah Dziadzio, Heather Mason-Suares, Hélène Cavé, Jennifer Lee, Karen Gripp, Kat Lafferty, Kezang Tshering, Lisa Vincent, Luis Enrique Gomez, Marco Tartaglia, Marina DiStefano, Martin Zenker, Reza Ahmadian, Ryan Webb, Shoji Ichikawa

## Conflict of Interest

Bradley Williams and Lisa M. Vincent work for a for-profit genetic testing center. Bruce D. Gelb receives royalties from GeneDx, Correlegan, LabCorp, and Prevention Genetics; consulted for Day One BioPharmaceuticals, BioMarin; and had sponsored research agreements from Day One BioPharmaceuticals, Onconova. All other authors declare no conflicts of interest.

## References

[1] Tidyman W.E., Rauen K.A. (2016). Pathogenetics of the RASopathies. Hum Mol Genet.

[2] Digilio M.C., Lepri F., Baban A. (2011). RASopathies: clinical diagnosis in the first year of life. Mol Syndromol.

[3] Amendola L.M., Jarvik G.P., Leo M.C. (2016). Performance of ACMG-AMP variant-interpretation guidelines among nine laboratories in the clinical sequencing exploratory research consortium. Am J Hum Genet.

[4] Seifert B.A., McGlaughon J.L., Jackson S.A. (2019). Determining the clinical validity of hereditary colorectal cancer and polyposis susceptibility genes using the Clinical Genome Resource Clinical Validity Framework. Genet Med.

[5] Rehm H.L., Berg J.S., Brooks L.D. (2015). ClinGen—the clinical genome resource. N Engl J Med.

[6] Strande N.T., Riggs E.R., Buchanan A.H. (2017). Evaluating the clinical validity of gene-disease associations: an evidence-based framework developed by the clinical genome resource. Am J Hum Genet.

[7] Richards S., Aziz N., Bale S. (2015). Standards and guidelines for the interpretation of sequence variants: a joint consensus recommendation of the American College of Medical Genetics and Genomics and the Association for Molecular Pathology. Genet Med.

[8] Rivera-Muñoz E.A., Milko L.V., Harrison S.M. (2018). ClinGen Variant Curation Expert Panel experiences and standardized processes for disease and gene-level specification of the ACMG/AMP guidelines for sequence variant interpretation. Hum Mutat.

[9] Grant A.R., Cushman B.J., Cavé H. (2018). Assessing the gene-disease association of 19 genes with the RASopathies using the ClinGen gene curation framework. Hum Mutat.

[10] Gelb B.D., Cavé H., Dillon M.W. (2018). ClinGen's RASopathy Expert Panel consensus methods for variant interpretation. Genet Med.

[11] Harrison S.M., Dolinsky J.S., Knight Johnson A.E. (2017). Clinical laboratories collaborate to resolve differences in variant interpretations submitted to ClinVar. Genet Med.

[12] Strande N.T., Brnich S.E., Roman T.S., Berg J.S. (2018). Navigating the nuances of clinical sequence variant interpretation in Mendelian disease. Genet Med.

[13] Harrison S.M., Biesecker L.G., Rehm H.L. (2019). Overview of specifications to the ACMG/AMP variant interpretation guidelines. Curr Protoc Hum Genet.

[14] McGlaughon J.L., Goldstein J.L., Thaxton C., Hemphill S.E., Berg J.S. (2018). The progression of the ClinGen gene clinical validity classification over time. Hum Mutat.

[15] Ioannidis N.M., Rothstein J.H., Pejaver V. (2016). REVEL: an ensemble method for predicting the pathogenicity of rare missense variants. Am J Hum Genet.

[16] Pejaver V., Byrne A.B., Feng B.J. (2022). Calibration of computational tools for missense variant pathogenicity classification and ClinGen recommendations for PP3/BP4 criteria. Am J Hum Genet.

[17] Wallace S.E., Bean L.J.H., Adam M.P., Feldman J., Mirzaa G.M. (2017). *GeneReviews®* [Internet].

[18] Kwon J.J., Hajian B., Bian Y. (2022). Structure-function analysis of the SHOC2-MRAS-PP1C holophosphatase complex. Nature.

[19] Bean L.J.H., Funke B., Carlston C.M. (2020). Diagnostic gene sequencing panels: from design to report-a technical standard of the American College of Medical Genetics and Genomics (ACMG). Genet Med.

[20] Roberts A.E., Allanson J.E., Tartaglia M., Gelb B.D. (2013). Noonan syndrome. Lancet.

[21] Hakami F., Dillon M.W., Lebo M., Mason-Suares H. (2016). Retrospective study of prenatal ultrasound findings in newborns with a Noonan spectrum disorder. Prenat Diagn.

[22] Lamouroux A., Dauge C., Wells C. (2022). Extending the prenatal Noonan’s phenotype by review of ultrasound and autopsy data. Prenat Diagn.

[23] Becker K., Hughes H., Howard K. (2007). Early fetal death associated with compound heterozygosity for Noonan syndrome-causative PTPN11 mutations. Am J Med Genet A.

[24] Brasil A.S., Malaquias A.C., Wanderley L.T. (2010). Co-occurring PTPN11 and SOS1 gene mutations in Noonan syndrome: does this predict a more severe phenotype?. Arq Bras Endocrinol Metab.

[25] Brnich S.E., Abou Tayoun A.N., Couch F.J. (2019). Recommendations for application of the functional evidence PS3/BS3 criterion using the ACMG/AMP sequence variant interpretation framework. Genome Med.

[26] Gremer L., Merbitz-Zahradnik T., Dvorsky R. (2011). Germline KRAS mutations cause aberrant biochemical and physical properties leading to developmental disorders. Hum Mutat.

[27] Cirstea I.C., Gremer L., Dvorsky R. (2013). Diverging gain-of-function mechanisms of two novel KRAS mutations associated with Noonan and cardio-facio-cutaneous syndromes. Hum Mol Genet.

[28] Rusyn E.V., Reynolds E.R., Shao H. (2000). Rit, a non-lipid-modified Ras-related protein, transforms NIH3T3 cells without activating the ERK, JNK, p38 MAPK or PI3K/Akt pathways. Oncogene.

[29] Umeki I., Niihori T., Abe T. (2019). Delineation of LZTR1 mutation-positive patients with Noonan syndrome and identification of LZTR1 binding to RAF1-PPP1CB complexes. Hum Genet.

[30] Walker L.C., Hoya Wiggins GAR. (2023). Using the ACMG/AMP framework to capture evidence related to predicted and observed impact on splicing: recommendations from the ClinGen SVI Splicing Subgroup. Am J Hum Genet.

[31] Wey M., Lee J., Jeong S.S., Kim J., Heo J. (2013). Kinetic mechanisms of mutation-dependent Harvey Ras activation and their relevance for the development of Costello syndrome. Biochemistry.

[32] Schubbert S., Bollag G., Lyubynska N. (2007). Biochemical and functional characterization of germ line KRAS mutations. Mol Cell Biol.

[33] Cirstea I.C., Kutsche K., Dvorsky R. (2010). A restricted spectrum of NRAS mutations causes Noonan syndrome. Nat Genet.

[34] Higgins E.M., Bos J.M., Mason-Suares H. (2017). Elucidation of MRAS-mediated Noonan syndrome with cardiac hypertrophy. JCI Insight.

[35] Johnston J.J., van der Smagt J.J., Rosenfeld J.A. (2018). Autosomal recessive Noonan syndrome associated with biallelic LZTR1 variants. Genet Med.

[36] Abou Tayoun A.N., Pesaran T., DiStefano M.T. (2018). Recommendations for interpreting the loss of function PVS1 ACMG/AMP variant criterion. Hum Mutat.

[37] Oza A.M., DiStefano M.T., Hemphill S.E. (2018). Expert specification of the ACMG/AMP variant interpretation guidelines for genetic hearing loss. Hum Mutat.

[38] Motta M., Fidan M., Bellacchio E. (2019). Dominant Noonan syndrome-causing LZTR1 mutations specifically affect the Kelch domain substrate-recognition surface and enhance RAS-MAPK signaling. Hum Mol Genet.

[39] Tartaglia M., Martinelli S., Stella L. (2006). Diversity and functional consequences of germline and somatic PTPN11 mutations in human disease. Am J Hum Genet.

[40] Mason-Suares H., Toledo D., Gekas J. (2017). Juvenile myelomonocytic leukemia-associated variants are associated with neo-natal lethal Noonan syndrome. Eur J Hum Genet.

[41] Malniece I., Grasmane A., Inashkina I. (2020). The fetal phenotype of Noonan syndrome caused by severe, cancer-related PTPN11 variants. Am J Case Rep.

[42] Kratz C.P., Niemeyer C.M., Castleberry R.P. (2005). The mutational spectrum of PTPN11 in juvenile myelomonocytic leukemia and Noonan syndrome/myeloproliferative disease. Blood.

[43] Walsh M.F., Ritter D.I., Kesserwan C. (2018). Integrating somatic variant data and biomarkers for germline variant classification in cancer predisposition genes. Hum Mutat.

