# Supplementary material for: Updated ACMG/AMP specifications for variant interpretation and gene curations from the ClinGen RASopathy expert panels
Source: Genet Med Open. 2025 Apr 17;3:103430. doi: 10.1016/j.gimo.2025.103430 (PMC12151217; doi:10.1016/j.gimo.2025.103430)
Supplement: Supplemental Material [file mmc1.pdf]

Supplementary Table 1. **RASopathy-associated gene general information, functional domains and hotspots**

RASopathy Gene	*BRAF*	*RAF1*	*MAP2K1*	*MAP2K2*	*PTPN11*	*SHOC2*	*SOS1*
**General Gene Information**							
Alternate/Alias Names	*BRAF1*	*RAF-1, C-RAF, CRAF*	*MEK1, PRKMK1, MAPKK1*	*MEK2, PRKMK2*	*NS1, BPTP3, SH-PTP2, SHP-2, PTP2C, SHP2*	*KIAA0862, SOC2, SUR-8, SOC-2, SUR8*	*GINGF, GF1*
NCBI Entrez Gene ID:	673	5894	5604	5605	5781	8036	6654
Primary Clinical and MANE Reference Sequences	NM_004333.4 MANE Select: NM_001374258.1 (NP_001361187.1)	NM_002880.3 MANE Select: NM_001354689.3 (NP_001341618.1)	NM_002755.3 MANE Select: NM_002755.4 (NP_002746.1)	NM_030662.3 MANE Select: NM_030662.4 (NP_109587.1)	NM_002834.3 MANE Select: NM_002834.5	NM_007373.3 MANE Select: NM_007373.4	NM_005633.4 (NP_005624.2)
PP2 applicable (gnomAD missense Z-score > 3.09)	Yes (Z = 3.72)	No (Z = 2.46)	Yes (Z = 3.11)	No (Z = 1.87)	Yes (Z = 3.13)	No (Z = 2.97)	No (Z = 3.05)
**Functional Domains and Mutation Hot Spot Analysis**							
Mutational Hot-Spot or Specific Amino Acid Residues at a Strong level (PM5_Strong)	W531	N/A	N/A	N/A	N308	N/A	M269, I437, R552
Specific Regions or Functional Domains Supporting Pathogenicity at a Moderate Level (PM1)	ex6, ex11, P-loop (aa 459-474); CR3 activation segment (aa 594-627)	CR2 domain [aa 251-266/ex7]; ex 14, ex 17	aa 43-61, 124-134	aa 47-65, 128-138	Directly interacting residues between N-SH2 and PTPN domains: aa 4, 7-9, 58-63, 69-77, 247, 251, 255, 256, 258, 261, 265, 278-281, 284.	N/A	N/A
References (PMID)	15488754 (note that AA redsides numbered incorrectly in article), 16439621, 17603483, 24957944, 15520807	19020799, 17603482, 17603483, 24957944, 9689060, 15520807	19156172, 22177953, 26399658, 25370473, 22753777	19156172; 22177953, 22753777; 26399658; 25370473	9491886, 16053901, 11992261		

RASopathy Gene	*SOS2*	*HRAS*	*KRAS*	*MRAS*	*NRAS*	*RIT1*	*RRAS2*	*LZTR1*	*PPP1CB*
**General Gene Information**									
Alternate/Alias Names	*NS9*	*HRAS1*	*KRAS2, KRAS1*	*NS11, RRAS3*	*NS6, CMNS, NCMS, ALPS4, NRAS1*	*RIT, RIBB, ROC1, NS8*	*TC21, NS12*	*LZTR-1, BTBD29, SWNTS2, NS2, NS10*	*PP1B, PP-1B, PP1beta, PP1c, MP, PPP1beta, NSLH2, PPP1CD, HEL-S-80p*
NCBI Entrez Gene ID:	6655	3265	3845	22808	4893	6016	22800	8216	5500
Primary Clinical and MANE Reference Sequences	NM_006939.4 (NP_008870.2)	NM_005343.3 (NP_005334.1) MANE Select: NM_005343.4	NM_004985.4 (NP_004976.2) MANE Select: NM_004985.5	NM_012219.4 (NP_036351.3) MANE Select: NM_001085049.3	NM_002524.4 (NP_002515.1) MANE Select: NM_002524.5	NM_006912.5 (NP_008843.1) MANE Select: NM_006912.6	NM_012250.5 (NP_036382.2) MANE Select: NM_012250.6	NM_006767.4 (NP_006758.2)	NM_206876.2 (NP_996759.1) MANE Select: NM_002709.3
PP2 applicable (gnomAD missense Z-score > 3.09)	No (Z = 2.53)	No (Z = 1.51)	No (Z = 2.32)	No (Z = 2.33)	No (Z = 1.71)	No (Z = 2.08)	No (Z=1.78)	No (Z = 0.58)	Yes (Z = 4.33)
**Functional Domains and Mutation Hot Spot Analysis**									
Mutational Hot-Spot or Specific Amino Acid Residues at a Strong level (PM5_Strong)	T264, M267	G12, G13, A59, G60, Q61	G12, G13, P34, A59, G60, Q61, D153	N/A	G12, G60	K23, A77, F82, M90	G23	N/A	N/A
Specific Regions or Functional Domains Supporting Pathogenicity at a Moderate Level (PM1)	N/A	P-loop, SW1, SW2, SAK	P-loop, SW1, SW2, SAK	P-loop, SW1, SW2 (no SAK)	P-loop, SW1, SW2, SAK	P-loop, SW1, SW2 (no SAK)	P-loop, SW1, SW2 (no SAK)	N/A	N/A
References (PMID)		24224811	17875937	28289718	28098151, 28594414, 19966803, 26467218	28554332, 26714497, 26757980, 27101134, 27109146, 29402968			

Supplementary Table 2. **RASopathy approved functional assays**

Assay	General Description	PS3 Criteria	Applicable Genes
RAS Activation Assay	Measures the bound RAS protein that immunoprecipitated with RAF1 or RBD (synthetic)	Increased RAS/RAF1 or RAS/RBD complexes	*MRAS, HRAS, KRAS, SOS1, SOS2, NRAS, LZTR1*
MEK Activation Assay	Measures the ratio of phosphorylated MEK to unphosphorylated MEK, basally and following RTK stimulation	Increased phosphorylation	*MRAS, BRAF, HRAS, KRAS, MAP2K1, MAP2K2, PTPN11, RAF1, SOS1, SOS2, NRAS, LZTR1*
ERK Activation Assay	Measures the ratio of phosphorylated ERK to unphosphorylated ERK, basally and following stimulation	Increased phosphorylation	*MRAS, BRAF, HRAS, KRAS, MAP2K1, MAP2K2, PTPN11, RAF1, SOS1, SOS2, NRAS, LZTR1*
SHP-2 Phosphatase Activity	Measures the ratio of phosphorylated and dephosphorylated SHP2	Increased dephosphorylation	*PTPN11*
BRAF Kinase Activity	Measures activity of kinase phosphorylating species of MEK and ERK in transfected cells	Increased phosphorylation	*BRAF*
RAF1 Kinase Activity	Measures activity of kinase phosphorylating species of MEK and ERK in transfected cells	Increased phosphorylation	*RAF1*
LZTR1 Stability Localization	Measures LZTR1 protein levels and location in transfected cells, basally and following stimulation	Decreased protein levels or abnormal localization within cells	*LZTR1*

```
SOS1 NP_005624.2 1     MQAQQLPYEFFSEENAPKWRGLLVPALKKVQGQVHPTLESNDDALQYVEELILQLLNMLCQAQPRSASDVEERVQKSFPH 80
SOS2 NP_008870.2 1     MQQAPQPYEFFSEENSPKWRGLLVSALRKVQEQVHPTLSANEESLYYIEELIFQLLNKLCMAQPRTVQDVEERVQKTFPH 80

SOS1 NP_005624.2 81    PIDKWAIADAQSAIEKRKRRNPLSLPVEKIHPLLKEVLGYKIDHQVSVYIVAVLEYISADILKLVGNYVRNIRHYEITKQ 160
SOS2 NP_008870.2 81    PIDKWAIADAQSAIEKRKRRNPLLLPVDKIHPSLKEVLGYKVDYHVSLYIVAVLEYISADILKLAGNYVFNIRHYEISQQ 160

SOS1 NP_005624.2 161   DIKVAMCADKVLMDMFHQDVEDINILSLTDEEPSTSGEQTYYDLVKAFMAEIRQYIRELNLIIKVFREPFVSNSKLFSAN 240
SOS2 NP_008870.2 161   DIKVSMCADKVLMDMFDQD--DIGLVSLCEDEPSSSGELNYYDLVRTEIAEERQYLRELNMIIKVFREAFLSDRKLFKPS 238

SOS1 NP_005624.2 241   DVENIFSRIVDIHELSVKLLGHIEDTVEMTDEGSPHPLVGSCFEDLAEELAFDPYESYARDILRPGFHDRFLSQLSKPGA 320
SOS2 NP_008870.2 239   DIEKIFSNISDIHELTVKLLGLIEDTVEMTDESSPHPLAGSCFEDLAEEQAFDPYETLSQDILSPEFHEHFNKLMARPAV 318

SOS1 NP_005624.2 321   ALYLQSIGEGFKEAVQYVLPRLLLAPVYHCLHYFELLKQLEEKSEDQEDKECLKQAITALLNVQSGMEKICSKSLAKRRL 400
SOS2 NP_008870.2 319   ALHFQSIADGFKEAVRYVLPRLMLVPVYHCWHYFELLKQLKACSEEQEDRECLNQAITALMNLQGSMDRIYKQYSPRRRP 398

SOS1 NP_005624.2 401   SESACRFYSQQMKGKQLAIKKMNEIQKNIDGWEGKDIGQCCNEFIMEGTLTRVGAKHERHIFLFDGLMICCKSNHGQPRL 480
SOS2 NP_008870.2 399   GDPVCPFYSHQLRSKHLAIKKMNEIQKNIDGWEGKDIGQCCNEFIMEGPLTRIGAKHERHIFLFDGLMISCKPNHGQTRL 478

SOS1 NP_005624.2 481   PGASNAEYRLKEKFFMRKVQINDKDDTNEYKHAFEIILKDENSVIFSAKSAEEKNNWMAALISLQYRSTLERMLDVTMLQ 560
SOS2 NP_008870.2 479   PGYSSAEYRLKEKFVMRKIQICDKEDTCEHKHAFELVSKDENSIIFAAKSAEEKNNWMAALISLHYRSTLDRMLDSVLLK 558

SOS1 NP_005624.2 561   EEKEEQMRLPSADVYRFAEPDSEENIIFEENMQPKAGIPIIKAGTVIKLIERLTYHMYADPNFVRTFLTTYRSFCKPQEL 640
SOS2 NP_008870.2 559   EENEQPLRLPSPEVYRFVVKDSEENIVFEDNLQSRSGIPIIKGGTVVKLIERLTYHMYADPNFVRTFLTTYRSFCKPQEL 638

SOS1 NP_005624.2 641   LSLIIERFEIPEPEPTEADRIAIENGDQPLSAELKRFRKEYIQPVQLRVLNVCRHWVEHHFYDFERDAYLLQRMEEFIGT 720
SOS2 NP_008870.2 639   LSLLIERFEIPEPEPTDADKLAIEKGEQPISADLKRFRKEYVQPVQLRILNVFRHWVEHHFYDFERDLELLERLESFISS 718

SOS1 NP_005624.2 721   VRGKAMKKWVESITKIIQRKKIARDNGPGHNITFQSSPPTVEWHISRPGHIETFDLLTLHPIEIARQLTLLESDLYRAVQ 800
SOS2 NP_008870.2 719   VRGKAMKKWVESIAKIIRRKKQAQANGVSHNITFESPPPPIEWHISKPGQFETFDLMTLHPIEIARQLTLLESDLYRKVQ 798

SOS1 NP_005624.2 801   PSELVGSVWTKEDKEINSPNLLKMIRHTTNLTLWFEKCIVETENLEERVAVVSRIIEILQVFQELNNFNGVLEVVSAMNS 880
SOS2 NP_008870.2 799   PSELVGSVWTKEDKEINSPNLLKMIRHTTNLTLWFEKCIVEAENFEERVAVLSRIIEILQVFQDLNNFNGVLEIVSAVNS 878

SOS1 NP_005624.2 881   SPVYRLDHTFEQIPSRQKKILEEAHELSEDHYKKYLAKLRSINPPCVPFFGIYLTNILKTEEGNPEVLKRHGKELINFSK 960
SOS2 NP_008870.2 879   VSVYRLDHTFEALQERKRKILDEAVELSQDHFKKYLVKLKSINPPCVPFFGIYLTNILKTEEGNNDFLKKKGKDLINFSK 958

SOS1 NP_005624.2 961   RRKVAEITGEIQQYQNQPYCLRVESDIKRFFENLNPMGNSMEKEFTDYLFNKSLEIEPRNPKPLPRFPKKYSYPLKSPGV 1040
SOS2 NP_008870.2 959   RRKVAEITGEIQQYQNQPYCLRIEPDMRRFFENLNPMGSASEKEFTDYLFNKSLEIEPRNCKQPPRFPRKSTFSLKSPGI 1038

SOS1 NP_005624.2 1041  RPSNPR----PGTMR-HPTPLQQEPRKISYSRIPESETESTASAPNSPRTPLTPPPASGASSTTDVCSVFDSDHSSPFHS 1115
SOS2 NP_008870.2 1039  RPNTGRHGSTSGTLRGHPTPLEREPCKISFSRIAETELESTVSAPTSPNTPSTPP----VSASSDLSVFLDVDLNS--SC 1112

SOS1 NP_005624.2 1116  SNDTVFIQVTLPHGPRSASVSSISLTKGTDEVPVPPPVPPRRRPESAPAESSPSKIMSKHLDSPPAIPPRQPTSKAYSPR 1195
SOS2 NP_008870.2 1113  GSNSIFAPVLLPHS-KSFFSSCGSLHKLSEEPLIPPPLPPRKKFDH---DASNSKGNMKSDDDPPAIPPRQPPPPKVKPR 1188

SOS1 NP_005624.2 1196  YSIS----DRTSISDPPESP----PLLPPREPVRTPDVFSSSPLHLQPPPLG---KKSD--HGNAFFPNSPSPFTPPPPQ 1262
SOS2 NP_008870.2 1189  VPVPTGAFDGPLHSPPPPPPRDPLPDTPPPVPLRPPEHFINCPFNLQPPPLGHLHRDSDWLRDISTCPNSPS----TPPS 1264

SOS1 NP_005624.2 1263  TPSPHGTRR-HLPSPPLTQEVDLHSIAGPPVPPRQSTSQHIPKLPPKTYKREHTHPSMHRDGPPLLENAHS-S 1333
SOS2 NP_008870.2 1265  TPSPRVPRRCYVLS---SSQNNLAHPPAPPVPPRQNSSPHLPKLPPKTYKRELSHPPLYR--LPLLENAETPQ 1332
```

Supplementary Figure 1: *SOS* alignment

Transcript NP_005624.2 of the *SOS1* gene. Transcript NP_008870.2 of the *SOS2* gene.

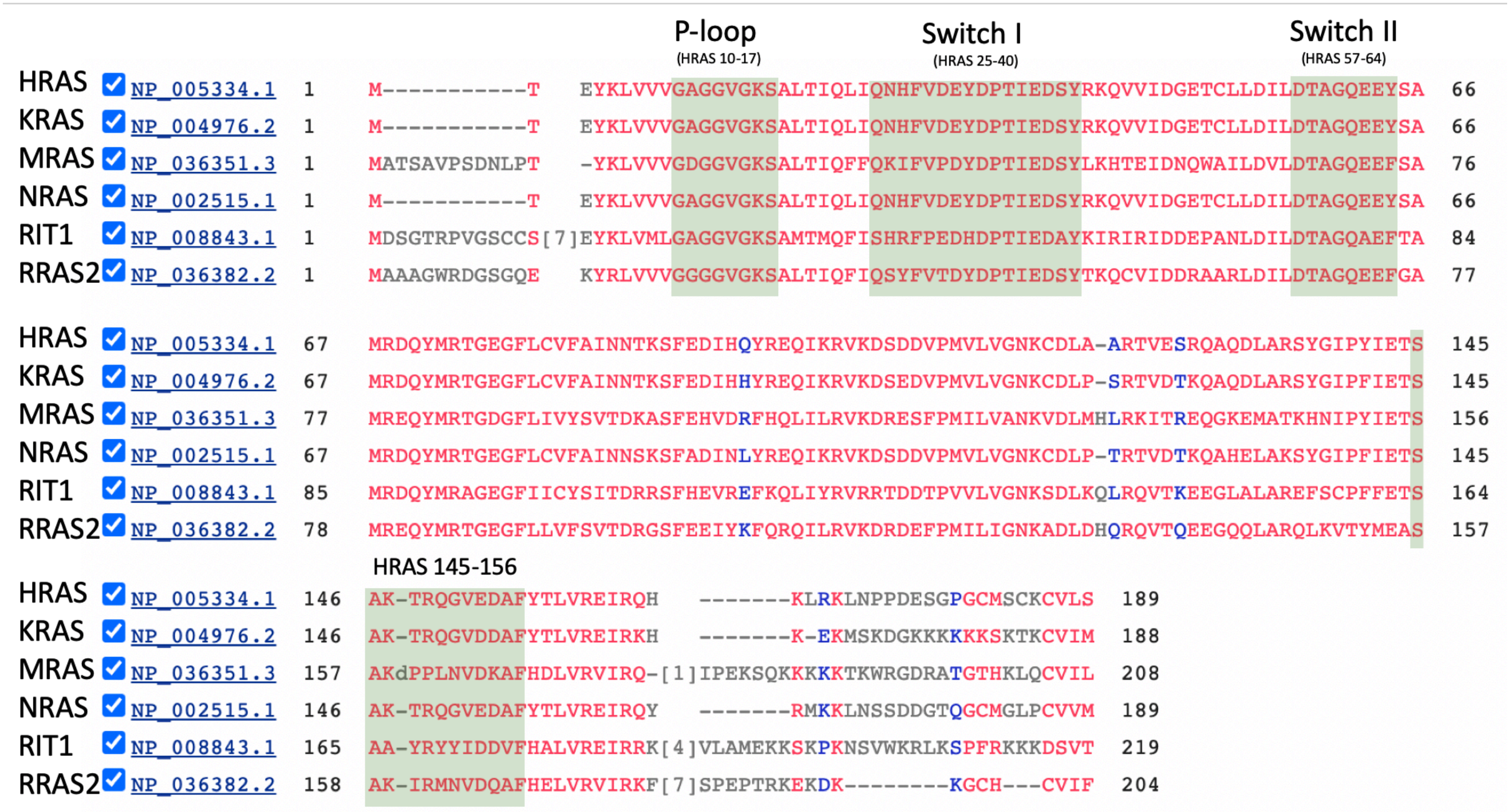

Supplementary Figure 2: ***RAS*** **alignment**

```
MAP2K1 1    -MPKKKPT--PIQLNPA-PDGSAVNGTSSAETNLEALQKKLEELELDEQQRKRLEAFLTQKQKVGELKDDDFEKISELGA  76
MAP2K2 1    MLARRKPVLPALTINPTIAEGPSPTSEGASEANLVDLQKKLEELELDEQQKKRLEAFLTQKAKVGELKDDDFERISELGA  80

MAP2K1 77   GNGGVVFKVSHKPSGLVMARKLIHLEIKPAIRNQIIRELQVLHECNSPYIVGFYGAFYSDGEISICMEHMDGGSLDQVLK  156
MAP2K2 81   GNGGVVTKVQHRPSGLIMARKLIHLEIKPAIRNQIIRELQVLHECNSPYIVGFYGAFYSDGEISICMEHMDGGSLDQVLK  160

MAP2K1 157  KAGRIPEQILGKVSIAVIKGLTYLREKHKIMHRDVKPSNILVNSRGEIKLCDFGVSGQLIDSMANSFVGTRSYMSPERLQ  236
MAP2K2 161  EAKRIPEEILGKVSIAVLRGLAYLREKHQIMHRDVKPSNILVNSRGEIKLCDFGVSGQLIDSMANSFVGTRSYMAPERLQ  240

MAP2K1 237  GTHYSVQSDIWSMGLSLVEMAVGRYPIPPPDAKELELMFGCQV----EGDAAETPPRPRTPGRPLSSYGMDSRPPMAIFE  312
MAP2K2 241  GTHYSVQSDIWSMGLSLVELAVGRYPIPPPDAKELEAIFGRPVVDGEEGEPHSISPRPRPPGRPVSGHGMDSRPAMAIFE  320

MAP2K1 313  LLDYIVNEPPPKLPSGVFSLEFQDFVNKCLIKNPAERADLKQLMVHAFIKRSDAEEVDFAGWLCSTIGLNQPSTPTHAAG  392
MAP2K2 321  LLDYIVNEPPPKLPNGVFTPDFQEFVNKCLIKNPAERADLKMLTNHTFIKRSEVEEVDFAGWLCKTLRLNQPGTPTRTA-  399

MAP2K1 393  V  393
MAP2K2 400  V  400
```

Supplementary Figure 3: ***MAP2K*** **alignment**

The first line is Transcript NP_002746.1 of the *MAP2K1* gene. The second line is the Transcript NP_109587.1

Of the *MAP2K2* gene.

```
BRAF  NP_001361187.1  1    MAALSGGGGGGAEPGQALFNGDMEPEAGAGAGAAASSAADPAIPEEVWNIKQMIKLTQEHIEALLDKFGGEHNPPSIYLE  80
RAF1  NP_001341618.1       --------------------------------------------------------------------------------

BRAF  NP_001361187.1  81   AYEEYTSKLDALQQREQQLLESLGNGTDFSVSSSASMDTVTSSSSSSLSVLPSSLSVFQNPTDVARSNPKSPQKPIVRVF  160
RAF1  NP_001341618.1  1    ------------MEHIQGAWKTISNGFGFK-------DAVFDGSSCISPTIVQQFGYQRRASDDGKLTDPSKTSNTIRVF  61

BRAF  NP_001361187.1  161  LPNKQRTVVPARCGVTVRDSLKKALMMRGLIPECCAVYRI---QDGEKKPIGWDTDISWLTGEELHVEVLENVPLTTHNF  237
RAF1  NP_001341618.1  62   LPNKQRTVVNVRNGMSLHDCLMKALKVRGLQPECCAVFRLLHEHKGKKARLDWNTDAASLIGEELQVDFLDHVPLTTHNF  141

BRAF  NP_001361187.1  238  VRKTFFTLAFCDFCRKLLFQGFRCQTCGYKFHQRCSTEVPLMCVNYDQLD--LLFVSKFFEHHPIPQEEASLAETALTSG  315
RAF1  NP_001341618.1  142  ARKTFLKLAFCDICQKFLLNGFRCQTCGYKFHEHCSTKVPTMCVDWSNIRQLLLFPNSTIGDSGVP---------ALPSL  212

BRAF  NP_001361187.1  316  SSPSAPASDSIGPQILTSPSPSKSIPIPQPFRPADEDHRNQFGQRDRSSSAPNVH-INTIEPVNIDDLIRDQGFRGDGAP  394
RAF1  NP_001341618.1  213  TMRRMRESVSRMP---VSSQHRYSTPHAFTFNTSSPSSEGSLSQRQRSTSTPNVHMVSTTLPVD-SRMIEN---------  279

BRAF  NP_001361187.1  395  LNQLMRCLRKYQSRTPSPLLHSVPSEIVFDFEPGPVFRGSTTGLSATPPASLPGSLTNVKALQ-KSPGP-QRERKSSSSS  472
RAF1  NP_001341618.1  280  -NNLSASPRAWSRR--------------FCLRGRDAIRSHSESASPSALSSSPNNLSPTGWSQPKTPVPAQRERAPVSGT  344

BRAF  NP_001361187.1  473  EDRNRMKTLGRRDSSDDWEIPDGQITVGQRIGSGSFGTVYKGKWHGDVAVKMLNVTAPTPQQLQAFKNEVGVLRKTRHVN  552
RAF1  NP_001341618.1  345  QEKNKIRPRGQRDSSYYWEIEASEVMLSTRIGSGSFGTVYKGKWHGDVAVKILKVVDPTPEQFQAFRNEVAVLRKTRHVN  424

BRAF  NP_001361187.1  553  ILLFMGYSTKPQLAIVTQWCEGSSLYHHLHIIETKFEMIKLIDIARQTAQGMDYLHAKSIIHRDLKSNNIFLHEDLTVKI  632
RAF1  NP_001341618.1  425  ILLFMGYMTKDNLAIVTQWCEGSSLYKHLHVQETKFQMFQLIDIARQTAQGMDYLHAKNIIHRDMKSNNIFLHEGLTVKI  504

BRAF  NP_001361187.1  633  GDFGLATVKSRWSGSHQFEQLSGSILWMAPEVIRMQDKNPYSFQSDVYAFGIVLYELMTGQLPYSNINNRDQIIFMVGRG  712
RAF1  NP_001341618.1  505  GDFGLATVKSRWSGSQQVEQPTGSVLWMAPEVIRMQDNNPFSFQSDVYSYGIVLYELMTGELPYSHINNRDQIIFMVGRG  584

BRAF  NP_001361187.1  713  YLSPDLSKVRSNCPKAMKRLMAECLKKKRDERPLFPQILASIELLARSLPKIHRSASEPSLNRAGFQTEDFSLYAC---A  789
RAF1  NP_001341618.1  585  YASPDLSKLYKNCPKAMKRLVADCVKKVKEERPLFPQILSSIELLQHSLPKINRSASEPSLHRAA-HTEDIN--ACTLTT  661

BRAF  NP_001361187.1  790  SPKTPIQAGGYGEFAAFK  807
RAF1  NP_001341618.1  662  SPRLPV-------F----  668
```

Supplementary Figure 4: *RAF* alignment

Transcript NP_001361187.1 of the *BRAF* gene. Transcript NP_001341618.1 of the *RAF1* gene.

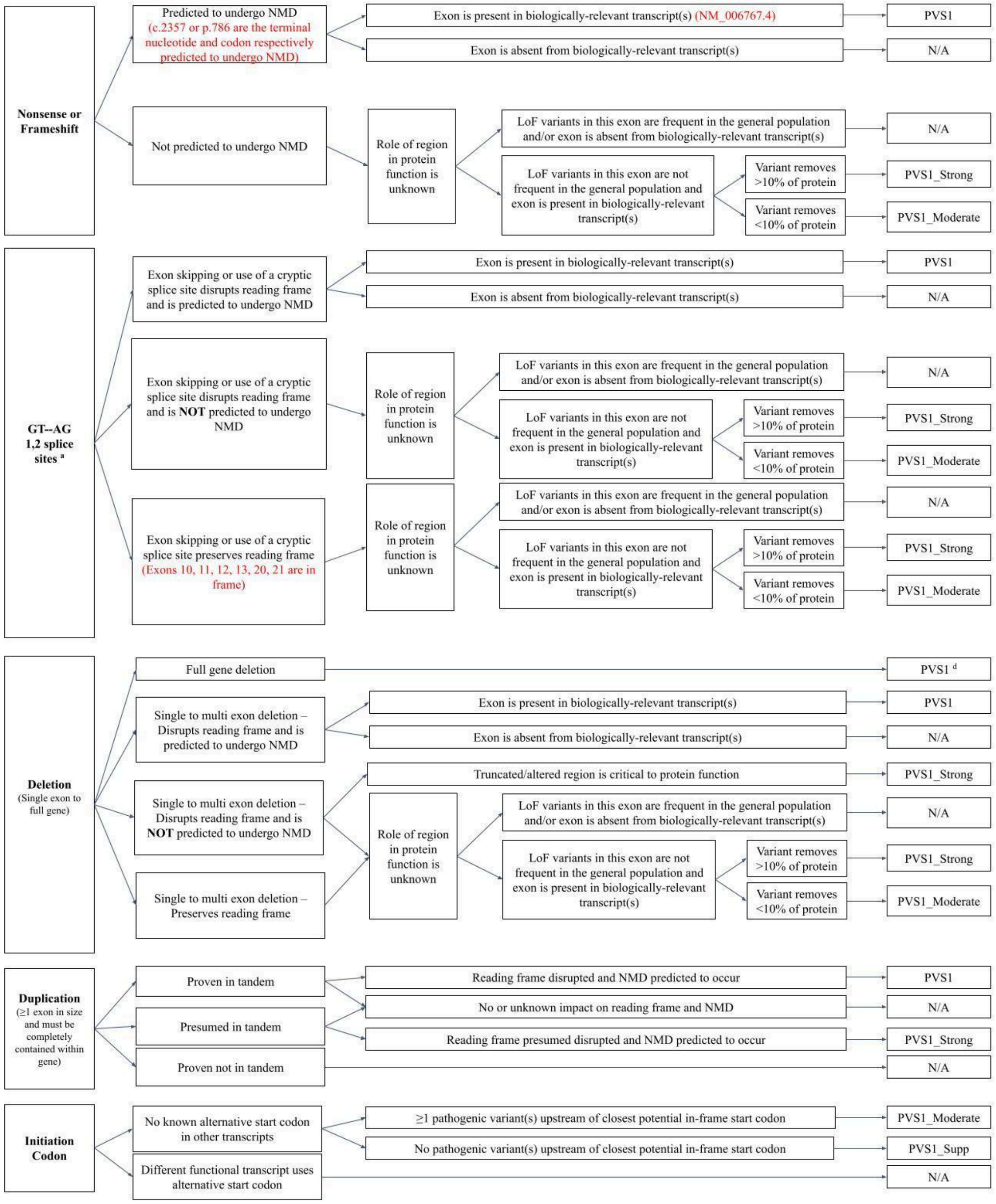

Nonsense or Frameshift
Predicted to undergo NMD (c.2357 or p.786 are the terminal nucleotide and codon respectively predicted to undergo NMD)
Exon is present in biologically-relevant transcript(s) (NM_006767.4)
PVS1
Exon is absent from biologically-relevant transcript(s)
N/A
Not predicted to undergo NMD
Role of region in protein function is unknown
LoF variants in this exon are frequent in the general population and/or exon is absent from biologically-relevant transcript(s)
N/A
LoF variants in this exon are not frequent in the general population and exon is present in biologically-relevant transcript(s)
Variant removes >10% of protein
PVS1_Strong
Variant removes <10% of protein
PVS1_Moderate
GT--AG 1,2 splice sites [a]
Exon skipping or use of a cryptic splice site disrupts reading frame and is predicted to undergo NMD
Exon is present in biologically-relevant transcript(s)
PVS1
Exon is absent from biologically-relevant transcript(s)
N/A
Exon skipping or use of a cryptic splice site disrupts reading frame and is NOT predicted to undergo NMD
Role of region in protein function is unknown
LoF variants in this exon are frequent in the general population and/or exon is absent from biologically-relevant transcript(s)
N/A
LoF variants in this exon are not frequent in the general population and exon is present in biologically-relevant transcript(s)
Variant removes >10% of protein
PVS1_Strong
Variant removes <10% of protein
PVS1_Moderate
Exon skipping or use of a cryptic splice site preserves reading frame (Exons 10, 11, 12, 13, 20, 21 are in frame)
Role of region in protein function is unknown
LoF variants in this exon are frequent in the general population and/or exon is absent from biologically-relevant transcript(s)
N/A
LoF variants in this exon are not frequent in the general population and exon is present in biologically-relevant transcript(s)
Variant removes >10% of protein
PVS1_Strong
Variant removes <10% of protein
PVS1_Moderate
Deletion (Single exon to full gene)
Full gene deletion
PVS1 [d]
Single to multi exon deletion – Disrupts reading frame and is predicted to undergo NMD
Exon is present in biologically-relevant transcript(s)
PVS1
Exon is absent from biologically-relevant transcript(s)
N/A
Single to multi exon deletion – Disrupts reading frame and is NOT predicted to undergo NMD
Truncated/altered region is critical to protein function
PVS1_Strong
Single to multi exon deletion – Preserves reading frame
Role of region in protein function is unknown
LoF variants in this exon are frequent in the general population and/or exon is absent from biologically-relevant transcript(s)
N/A
LoF variants in this exon are not frequent in the general population and exon is present in biologically-relevant transcript(s)
Variant removes >10% of protein
PVS1_Strong
Variant removes <10% of protein
PVS1_Moderate
Duplication (≥1 exon in size and must be completely contained within gene)
Proven in tandem
Presumed in tandem
Proven not in tandem
Reading frame disrupted and NMD predicted to occur
PVS1
No or unknown impact on reading frame and NMD
N/A
Reading frame presumed disrupted and NMD predicted to occur
PVS1_Strong
N/A
Initiation Codon
No known alternative start codon in other transcripts
≥1 pathogenic variant(s) upstream of closest potential in-frame start codon
PVS1_Moderate
No pathogenic variant(s) upstream of closest potential in-frame start codon
PVS1_Supp
Different functional transcript uses alternative start codon
N/A

Supplementary Figure 5: *LZTR1* PVS1 Decision Tree

This is only applicable to variants in *LZTR1.*
